# Antimicrobial Activity of Sorghum Phenolic Extract on Bovine Foodborne and Mastitis-Causing Pathogens

**DOI:** 10.3390/antibiotics10050594

**Published:** 2021-05-17

**Authors:** Sydney E. Schnur, Raghavendra G. Amachawadi, Giovanna Baca, Sarah Sexton-Bowser, Davina H. Rhodes, Dmitriy Smolensky, Thomas J. Herald, Ramasamy Perumal, Daniel U. Thomson, Tiruvoor G. Nagaraja

**Affiliations:** 1 Department of Diagnostic Medicine/Pathobiology, Kansas State University, Manhattan, KS 66506, USA; sydney59@vet.ksu.edu (S.E.S.); gbaca@vet.ksu.edu (G.B.); tnagaraj@vet.ksu.edu (T.G.N.); 2 Department of Clinical Sciences, Kansas State University, Manhattan, KS 66506, USA; 3 Center for Outcomes Research and Epidemiology, College of Veterinary Medicine, Kansas State University, Manhattan, KS 66502, USA; 4 Department of Agronomy, Kansas State University, Manhattan, KS 66506, USA; sarahann@ksu.edu; 5 Department of Horticulture & Landscape Architecture, Colorado State University, Fort Collins, CO 80521, USA; davina.rhodes@colostate.edu; 6 Center for Grain and Animal Health Research, USDA, Manhattan, KS 66502, USA; dmitriy.smolensky@usda.gov (D.S.); thomasherald98@gmail.com (T.J.H.); 7 Agriculture Research Center, Kansas State University, Hays, KS 67601, USA; perumal@ksu.edu; 8 Department of Animal Sciences, Iowa State University, Ames, IA 50011, USA; thomson1@iastate.edu

**Keywords:** antibacterial activity, foodborne pathogens, mastitis pathogens, sorghum grain extract

## Abstract

Antimicrobial resistance in bacterial pathogens associated with bovine mastitis and human foodborne illnesses from contaminated food and water have an impact on animal and human health. Phenolic compounds have antimicrobial properties and some specialty sorghum grains are high in phenolic compounds, and the grain extract may have the potential as a natural antimicrobial alternative. The study’s objective was to determine antimicrobial effects of sorghum phenolic extract on bacterial pathogens that cause bovine mastitis and human foodborne illnesses. Bacterial pathogens tested included *Escherichia coli*, *Salmonella* Typhimurium, *Campylobacter jejuni*, *Campylobacter coli*, *Pseudomonas aeruginosa*, *Klebsiella pneumoniae*, *Klebsiella oxytoca*, *Staphylococcus aureus*, and *Enterococcus faecalis*. Antibacterial activities of sorghum phenolic extracts were determined by agar-well diffusion assay. Sorghum phenolic extract was added to the wells in concentrations of 0, 100, 200, 500, 1000, or 4000 µg/mL. The control wells did not receive phenolic extract. Plates were incubated for 18–24 h, and the diameter of each zone of inhibition was measured. The results indicated that sorghum phenolic extract had inhibitory effects on *Staphylococcus aureus*, *Enterococcus faecalis*, *Campylobacter jejuni*, and *Campylobacter coli*.

## 1. Introduction

Increased antimicrobial resistance due to agricultural use has become a major issue around the world [1]. An increase in resistance to medically important antimicrobials, such as macrolides, third-generation and higher cephalosporins, fluoroquinolones, tetracyclines, and glycopeptides, led to increased scrutiny in the use of antibiotics in food animals with emphasis on antibiotic stewardship [2,3,4].

Zoonotic foodborne illnesses are a major public health concern, and food-producing animals are a major reservoir for many of the foodborne pathogens. The three major foodborne bacterial pathogens that food animals harbor are Shiga toxin-producing *Escherichia coli*, *Salmonella enterica*, and *Campylobacter* species, particularly *jejuni* and *coli*. The Center for Disease Controls recognizes the threat of these pathogens to One Health, spanning both human, animals, and the environment [5]. Brown et al. reported that 21% of 176 *Salmonella* foodborne illness outbreaks in the United Stated between 2003 and 2012 were resistant to at least one antibiotic [6]. Furthermore, Europe had 319 foodborne outbreaks linked to *Campylobacter* in 2019, which was 23% of the total number of outbreaks caused by bacteria in Europe [7]. Likewise, *Campylobacter* has been related to foodborne illness outbreaks in the United States and China [8,9]. In 2017, the CDC reported 23 Campylobacter outbreaks, 4% of the total number of outbreaks caused by bacteria in the United States [10]. Furthermore, illnesses involving *E. coli* O157 can lead to hemolytic uremic syndrome [11]. In 2019, 42 Shiga toxin-producing *E. coli* outbreaks were reported in Europe, which was 3% of the total number of outbreaks caused by bacteria in Europe [7]. Likewise, the United States reported that Shiga toxin-producing *E. coli* caused 3% of the total number of bacterial outbreaks in 2017 [10]. Hemolytic uremic syndrome (HUS) is a severe illness involving the kidneys that leads to acute renal failure and is more often seen in children and immunocompromised adults [12,13]. Furthermore, 87,923 human salmonellosis cases were reported in Europe in 2019 [7]. Antimicrobial resistance coupled with foodborne illness outbreaks among *Campylobacter*, *Escherichia coli* O157, and *Salmonella enterica* are a growing public health threat [6,14,15].

Furthermore, mastitis is an important production disease of dairy cattle. Mastitis is typically caused by various bacterial pathogens, such as *Staphylococcus aureus*, *Pseudomonas aeruginosa*, and *Klebsiella,* and causes reduced milk yield and economic losses to producers [16,17,18,19]. Ohnishi et al. [20] demonstrated that mastitis cases associated with *P. aeruginosa* can lead to acute systemic infections that are moderate to severe. Additionally, *P. aeruginosa* is known to be multidrug-resistant and, therefore, difficult to treat [20,21,22,23]. Tenhagen et al. [24] reported that 631 isolates of *S. aureus* from various cattle production areas were resistant to at least one antimicrobial. Isolates of *Escherichia coli*, *Klebsiella pneumoniae*, and *Pseudomonas aeruginosa* have also shown resistance to antimicrobials [25]. These findings demonstrate the need to find antimicrobial alternatives for food animal medicine, either for treatment or for prevention of diseases.

Antimicrobial alternatives are substitutions to existing antibiotics where bacterial resistance has emerged [26]. There are three major categories of antimicrobial alternatives: natural, synthetic, and biotechnology-based [27]. Plant-based antimicrobial compounds are part of the naturally occurring antimicrobial alternative category [27,28]. These compounds are typically secondary metabolites derived from plants with antioxidant and antimicrobial activity [29,30]. The mode of actions for antimicrobial activity to combat these pathogens are believed to be the reduction of pH, disruption of the permeability of the bacterial membrane, or through manipulation of bacterial efflux pumps [29].

Sorghum phenolic extract is a collection of plant-based compounds and a potent natural antimicrobial alternative [31,32]. Likewise, it has been shown to have many health and pharmaceutical benefits [33]. Genetic diversity offers some sorghum specialty lines with high tannin content and/or high phenolic compounds that demonstrate high antioxidant activity [34]. Also, the increased concentrations of phenolic compounds have been associated with higher antioxidant activities, anticarcinogenic effects, and antimicrobial activities [31,35,36]. Kil et al. [31] reported that a cultivar of sorghum phenolic extract had minimum inhibitory concentrations (MIC) for *S. aureus*, *Bacillus subtilis*, *Salmonella* Typhimurium, *K. pneumoniae*, and *E. coli* of 250, 250, 500, 500, and 250 µg/mL, respectively. Furthermore, Villalobos et al. [32] demonstrated antimicrobial activity of soy flour phenolic extract against *Listeria innocua*, *Listeria monocytogenes*, *Staphylococcus aureus*, *Bacillus cereus*, and *Enterococcus faecalis*. Additionally, a sorghum extract with phenol present in high amount has been shown to attenuate replication of *Legionella pneumophila* in mouse macrophage cells [37].

The current study’s objective was to investigate the antimicrobial activity of phenolic compounds extracted from sorghum bran against foodborne and mastitis-causing pathogens associated with cattle. We hypothesized that extracts from high phenolic sorghum grain would significantly inhibit pathogens in a dose-dependent manner.

## 2. Results

### 2.1. Foodborne Pathogens

The sorghum phenolic extract did not inhibit *E. coli* O157:H7 and *S*. Typhimurium but inhibited *C. jejuni* and *C. coli* (*p* ≤ 0.0001; Table 1).

The average zones of inhibition demonstrated for *C. jejuni* at concentrations of 0 µg/mL (control), 100 µg/mL, 200 µg/mL, 500 µg/mL, 1000 µg/mL, and 4000 µg/mL were 0.7, 1.9, 4.6, 6.8, and 13.2 mm, respectively. The zone of inhibition with different concentrations did differ significantly (*p* ≤ 0.0001; Table 1 and Table 2). The average zones of inhibition against *C. coli* at concentrations of 0 µg/mL (control), 100 µg/mL, 200 µg/mL, 500 µg/mL, 1000 µg/mL, and 4000 µg/mL were 0.0, 1.2, 2.4, 4.1, 6.8, and 13.0 mm, respectively. The concentration effect on zone of inhibition was significant (*p* < 0.05; Table 1 and Table 2).

### 2.2. Mastitic Pathogens

Overall, sorghum phenolic extract did not have inhibitory effects on *P. aeruginosa*, *K. pneumoniae*, and *K. oxytoca* (Table 1). However, significant inhibitory effects were observed between different concentrations of sorghum phenolic extract against *P. aeruginosa*, *K. pneumoniae*, and *K. oxytoca* (Table 1 and Table 2). There were statistical differences seen for the following comparisons of within and between sorghum phenolic extract concentrations when tested against *P. aeruginosa* and *K. pneumoniae* (*p* < 0.0001). The concentrations of sorghum phenolic extract with the largest zones of inhibition when used against *K. pneumoniae* were 100 µg/mL and 200 µg/mL, both with zones of inhibition of 1.3 mm. The zones of inhibition from sorghum phenolic extract tested against *K. oxytoca* demonstrated statistical differences for the following concentration comparisons: 0 µg/mL (control) vs. 1000 µg/mL, 100 µg/mL vs. 4000 µg/mL, 200 µg/mL vs. 4000 µg/mL, and 500 µg/mL vs. 4000 (*p* = 0.05). The concentration that sorghum phenolic extract had the greatest zone of inhibition for *K. oxytoca* was 4000 µg/mL, with a zone of inhibition of 2.4 mm.

The overall inhibitory effect of sorghum phenolic extract was significant when tested against *Staphylococcus aureus* (*p* ≤ 0.0001; Table 1). The average zones of inhibition recorded for *S. aureus* at various concentrations of 0 µg/mL (control), 100 µg/mL, 200 µg/mL, 500 µg/mL, 1000 µg/mL, and 4000 µg/mL were 0.0, 2.8, 3.4, 4.1, 5.2, and 7.7 mm, respectively, which was significant when tested between concentrations (*p* < 0.05). When compared between sorghum phenolic extract concentrations, all comparisons had *p-*values less than 0.05 (Table 2 and Table 3).

### 2.3. Opportunistic Commensal Bacteria

The overall inhibitory effect of sorghum phenolic extract was significant when tested against *Enterococcus faecalis* (*p* ≤ 0.0001; Table 1). The average zones of inhibition demonstrated when sorghum phenolic extract was tested against *E. faecalis* at concentrations of 0 µg/mL (control), 100 µg/mL, 200 µg/mL, 500 µg/mL, 1000 µg/mL, and 4000 µg/mL were 0.0, 1.7, 2.3, 3.0, 4.3, and 8.2 mm, respectively. The zone of inhibition in comparison with the various tested concentrations did differ significantly (*p* ≤ 0.05; Table 2 and Table 3).

### 2.4. Minimum Inhibitory Concentrations of Antibiotics and Sorghum Phenolic Compounds

MIC was performed for the two most commonly used antibiotics in cattle production systems for prophylaxis and therapeutic purposes. Antimicrobial susceptibility testing was done for 4 bacterial isolates (*Enterococcus faecalis*, *Staphylococcus aureus*, *Campylobacter coli* and *C. jejuni*), which showed weak-to-moderate effects with sorghum phenolic compound. *Campylobacter coli* and *C. jejuni* isolates were susceptible to erythromycin and tetracycline antibiotics. The *C. coli* isolate had an MIC value of 6.25 µg/mL and 1.56 µg/mL for erythromycin and tetracycline, respectively. The *C. jejuni* isolate had an MIC value of 3.12 µg/mL and 1.56 µg/mL for erythromycin and tetracycline, respectively. Both *E. faecalis* and *S. aureus* isolates were resistant to erythromycin and tetracycline antibiotics (>100 µg/mL). Consistent with the well diffusion assay, the sorghum phenolic compound was found inhibitory against all four bacteria by microbroth dilution procedure with an MIC value of 12.5 µg/mL, 12.5 µg/mL, 12.5 µg/mL, and 6.25 µg/mL for *Campylobacter coli, C. jejuni, E. faecalis* and *S. aureus* isolates, respectively.

## 3. Discussion

**Figure 1:**
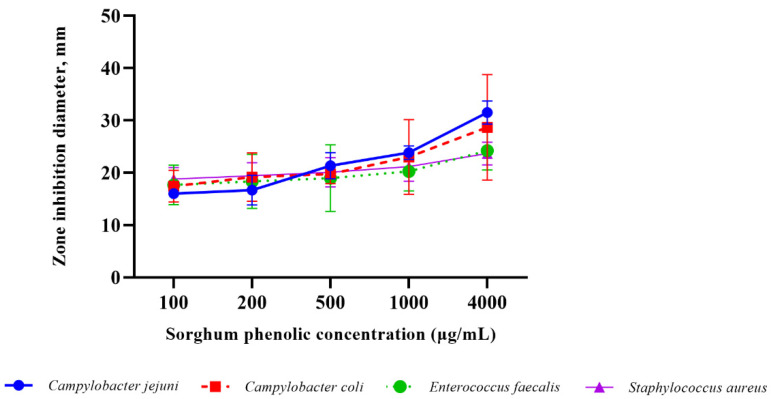
Antibacterial effects of sorghum phenolic extract concentrations for *Campylobacter jejuni*, *Campylobacter coli*, *Enterococcus faecalis*, and *Staphylococcus aureus*. Each line represents an average value (of three separate runs) with 95% CI.

Antimicrobial alternatives to control foodborne illness and bovine mastitis-causing pathogens are crucial for researchers to explore. Foodborne illness outbreaks caused by *Escherichia coli* O157, *Salmonella* Typhimurium, and *Campylobacter* cause economic impacts on communities due to decreased worker production and, in some cases, they can cause serious disease capable of resulting in kidney failure and death [11,38,39,40]. Furthermore, mastitis causes economic losses to milk producers and is a major health concern for lactating cows [20,41]. Our findings suggest that sorghum phenolic extract has antimicrobial effects against *Campylobacter*, but not *E. coli* O157 or *S*. Typhimurium. We also observed antimicrobial effects against *Staphylococcus aureus*, a Gram-positive mastitis-causing bacterium, and *Enterococcus faecalis*, a Gram-positive bacterium, a major nosocomial pathogen, particularly in severely immunocompromised individuals. Sorghum phenolic extract had no observed antimicrobial effect on Gram-negative bacteria studied, except for *Campylobacter*, while it had antimicrobial effects on both Gram-positive bacteria tested. *Campylobacter* is a microaerophilic organism, and this could explain why it was sensitive to the sorghum phenolic extract. Other high antioxidant compounds have been demonstrated to inhibit *Campylobacter* as well [42,43]. For example, Duarte et al. [42] reported coriander essential oil and linalool to have zones of inhibition that were about 85 mm. Resistance of the rest of the Gram-negative bacteria could be due to their lipopolysaccharide outer member protecting them from penetration of sorghum phenolic extract [32]. A limitation of this study was that the screening of sorghum was not exhaustive and instead represents a screening of sorghums with known high phenolics. Questions of which specific compound(s) in sorghums has efficacy, and the representation of those compound(s) in the diversity of sorghum was not in the scope of this project. Furthermore, a second limitation was that only two species of Gram-positive bacteria were tested, but the findings are similar to other researchers [32,44]. Akogou et al. [44] demonstrated no antimicrobial activity of red, phytoalexin-rich sorghum extract against *E. coli* O157. Likewise, Villalobos et al. [32] demonstrated that Gram-positive bacteria were more susceptible to natural phenolic extract from soybeans. However, the previous authors did show inhibitory effects on *E. coli* and *Salmonella* at 1250 and 1500 µg/mL [32]. Furthermore, Kil et al. [31] demonstrated sorghum phenolic extract to have minimum inhibitory concentrations of less than 1000 µg/mL for *S. aureus*, *Salmonella* Typhimurium, *Klebsiella pneumonia*, and *Escherichia coli* for four different sorghum lines. These results are different than our findings but could be due to different methods used to determine antimicrobial activity or, more likely, differences in the sorghum cultivars used. Furthermore, an upward dose-dependent trend of antimicrobial activity was seen for *C. jejuni, C. coli*, *E. faecalis*, and *S. aureus* with 100 ug/mL having the lowest zone of inhibition and 4000 ug/mL having the highest zone of inhibition (Figure 1).

Apart from *Campylobacter*, Gram-negative foodborne illness-causing bacteria used in this study were not inhibited by sorghum phenolic extract. However, sorghum grain could still be used as an antimicrobial alternative to these bacteria. Kumar et al. [45] engineered silver glyconanoparticles using sweet sorghum syrup that were shown to have antimicrobial effects on *E. coli*, *P. aeruginosa*, and *Klebsiella planticola*, as well as some Gram-positive bacteria. Furthermore, Halder et al. [46] identified a glycine and proline-rich protein in sorghum that has demonstrated antimicrobial activity against *Bacillus subtilis*, *Rhodococcus fascians*, and *Escherichia coli* with minimum inhibitory concentrations of 7.5, 22.4, and 75 µg/mL, respectively. Together, these findings demonstrate how sorghum grain can be used in different ways to develop antimicrobial alternatives and how further research should be conducted on sorghum antimicrobial effects.

## 4. Materials and Methods

### 4.1. Sorghum Plant Material

The diversified sorghum association panel (SAP) was evaluated at Manhattan, KS, and at Puerto Vallarta, Mexico, in 2014 along with other photosensitive lines. All lines were tested for total phenolic compounds concentrations and oxygen radical absorption capacity (ORAC) values. One photosensitive line PI570481 was chosen for the study as this line had ORAC values greater than 150 µM Trolex equivalent. The line PI570481 is a black-grained sorghum, selected due to its efficacy and high phenolic content, as previously published [37,47].

### 4.2. Total Phenolic Compounds Extraction and Quantification

A tangential abrasive dehulling device (Venables Machine Works, Saskatoon, SK, Canada) was used to remove the bran. Twenty-five mL of acidified methanol (1% HCl/methanol *v*/*v*) were used to suspend 0.5 g of the bran sample, which was then shaken for two hours. The mixture was centrifuged for 15 min and the supernatant was transferred into 50 mL tubes. Total phenols in the sorghum bran extract were determined using the method described by Herald et al. [34]. Seventy-five μL of water were added to each well in a 96-well plate. Twenty-five µL of sample extract, Trolox standard (6-Hydroxy-2,5,7,8-tetramethylchromane-2-carboxylic acid), or blank, along with 25 µL Folin-Ciocalteu reagent, were then added to each well and allowed to equilibrate at room temperature for 6 min. After equilibrating, 100 µL of 7.5% Na_2_CO_3_ were added to each well. The plate was placed at room temperature and left in the dark for 90 min. The absorbance was measured with a Synergy 2 microplate reader (BioTek, Winooski, VT, USA) at 765 nm and reported as mg gallic acid equivalents (GAE)/g of sorghum bran. Four replicates were used to measure each sample and the extraction resulted in phenolic extract with 4.0–4.4 mg/mL concentration of phenols.

### 4.3. Antimicrobial Susceptibility Testing of Sorghum Phenolic Extract

#### 4.3.1. Preparation of Bacterial Inocula

*Staphylococcus aureus* VDL3-SA-2017, *Pseudomonas aeruginosa* VDL4-PA-2017, *Klebsiella pneumoniae* VDL1-KP-2017, and *Klebsiella oxytoca* VDL2-KO-2017 isolated from milk samples from bovine at the Kansas State Veterinary Diagnostic Laboratory, *Escherichia coli* O157:H7 (2017-5-590), *Escherichia coli* O157:H7 (2017-5-493), and *Escherichia coli* O157:H7 (380-94) isolated from bovine feces, and *Enterococcus faecalis* ATCC 29212 were streaked onto blood agar plates (Remel Inc., Lenexa, KS, USA) and incubated at 37 °C for 24 h. *Campylobacter jejuni* and *Campylobacter coli* were streaked onto Mueller-Hinton (MH) agar (Becton Dickinson, Sparks, MD, USA) and incubated at 37 °C for 24 h in a tri-gas incubator under a microaerophilic condition. A single colony of each species from the plates was then suspended into 10 mL of phosphate buffer saline (PBS; Becton Dickinson, Sparks, MD, USA) until a turbidity equivalent to 0.5 McFarland standard was achieved per CLSI guidelines [48].

#### 4.3.2. Agar-Well Diffusion Assay

The antimicrobial activities of the sorghum phenolic extract were determined by a well diffusion assay [49]. Sixteen mm diameter wells were punched into MH agar (Becton Dickinson) and a small amount (approx. 30 µL) of melted MH agar was used to seal the bottom of the wells. A cotton swab was used to inoculate bacterial inoculum of the bacterial species to be tested onto MH agar with wells to obtain a lawn of growth. The wells were then filled with 100 µL of sorghum phenolic extract at concentrations of 0 (control), 100, 200, 500, 1000, or 4000 µg/mL with LB broth. Seventy percent ethanol was used to dissolve the sorghum phenolic extract and was added to wells as negative controls. The plates were incubated at 37 °C for 24 h aerobically or under microaerophilic conditions (for *Campylobacter* spp.). The diameter of the inhibitory zone was recorded.

### 4.4. Antibiotic Susceptibility Determinations

Minimum inhibitory concentrations of antibiotics were determined by the micro-broth dilution method [48]. Antibiotics tested were erythromycin and tetracycline (Sigma-Aldrich, St. Louis, MO, USA). Antibiotic stock solutions were prepared as per manufacturer’s guidelines to obtain a concentration of 1000 µg/mL based on potency of antibiotics. Antibiotics were tested at concentrations of 100, 50, 25, 12.5, 6.25, 3.125, 1.56, 0.78, 0.39, 0.195, and 0.098 µg/mL. The bacterial inocula were prepared by preparing 1:100 dilutions of cultures grown in 10 mL Mueller Hinton broth for 6 to 8 h. The antimicrobial susceptibility was performed in 96-well micro titer plates (Becton and Dickinson, Franklin Lakes, NJ, USA). Plates were incubated at 37 °C for 20–24 h under aerobic (*Enterococcus faecalis* and *Staphylococcus aureus*) and or microaerophilic conditions (*Campylobacter coli and C. jejuni*) and results were recorded as growth or no growth. The procedure was repeated again with different bacterial inoculum. As a reference standard, sorghum phenolic compound was also subjected for testing at the same concentration like antibiotics.

### 4.5. Statistical Analysis

The inhibitory activity of sorghum phenolic extract against foodborne illness and mastitis-causing bacterial pathogens was evaluated using a PROC MIXED procedure in SAS (Version 9.4, Cary, NC, USA). The statistical model included the concentrations of phenolic extract, replications (repeated measures as a random effect) and zone diameter of well diffusion assay. For the MIC data, values with >100 µg/mL were not analyzed. The mean comparisons within and between bacterial isolates against various concentrations of phenolic extract were tested using Tukey’s Honest Significant Difference with an alpha of ≤0.05.

## 5. Conclusions

Our data demonstrated that phenolic extract of a sorghum cultivar exhibited antibacterial activity. Of the mastitis-causing pathogens and foodborne pathogens prevalent in cattle tested, only *S. aureus*, *E. faecalis*, *C. jejuni*, and *C. coli* were inhibited by the phenolic extracts in a dose-dependent manner. The selective inhibition of certain bacterial species, both Gram-positive and Gram-negative bacteria, is an interesting observation that needs to be investigated. Our study provides impetus to test additional cultivars of phenolic acid extracts of sorghum cultivars and explore value-added benefits of sorghum grains as natural antimicrobial compounds against bacterial pathogens.

## Figures and Tables

**Table 1:** Overall antimicrobial effects of sorghum phenolic extract.

	Sorghum Phenolic Extract Concentration (µg/mL)						*p-*Value
	0	100	200	500	1000	4000	
Bacterial Species and Strain Number	ZOI ^a^	ZOI ^a^	ZOI ^a^	ZOI ^a^	ZOI ^a^	ZOI ^a^	
*Escherichia coli* O157 2017-5-590	0.0	0.0	0.0	0.0	0.0	0.0	-
*Escherichia coli* O157 2017-5-493	0.0	0.0	0.0	0.0	0.0	0.0	-
*Escherichia coli* O157 380-94	0.0	0.0	0.0	0.0	0.0	0.0	-
*Campylobacter jejuni* 2016-12-17A	0.0	0.0	0.0	4.1	5.8	11.1	0.0002
*Campylobacter coli* 2016-12-82A	0.0	1.4	3.2	3.8	7.0	12.7	0.0016
*Campylobacter coli* 2016-12-80A	0.0	0.0	0.8	3.8	5.8	11.7	<0.0001
*Campylobacter coli* 2016-12-181A	0.0	2.3	3.3	4.8	7.7	14.7	0.0004
*Salmonella* Typhimurium ATCC 14028	0.0	0.0	0.0	0.0	0.0	0.0	-
*Staphylococcus aureus* VDL3-SA-2017	0.0	2.8	3.4	4.1	5.2	7.7	0.0001
*Pseudomonas aeruginosa* VDL4-PA-2017	0	0.0	0.0	0.0	0.0	0.3	-
*Klebsiella pneumoniae* VDL1-KP-2017	0	1.3	1.3	0.9	0.0	0.8	0.8887
*Klebsiella oxytoca* VDL2-KO-2017	0.0	0.0	0.0	0.0	0.8	2.4	-
*Enterococcus faecalis* ATCC 29212	0.0	1.7	2.3	3.0	4.3	8.2	0.0003

^a^ ZOI: average zone of inhibition (diameter; mm).

**Table 2:** Antimicrobial effects of different sorghum phenolic extract concentrations.

	Sorghum Phenolic Extract Concentration (µg/mL)					Mean Differences with the *p*-Values																			
	100	200	500	1000	4000	100.vs. 200		100.vs. 500		100.vs. 1000		100.vs. 4000		200.vs. 500		200.vs. 1000		200.vs. 4000		500.vs. 1000		500.vs. 4000		1000.vs. 4000	
Bacteria	ZOI ^a^	ZOI ^a^	ZOI ^a^	ZOI ^a^	ZOI ^a^	SEM ^b^	*p*	SEM ^b^	*p*	SEM ^b^	*p*	SEM ^b^	*p*	SEM ^b^	*p*	SEM ^b^	*p*	SEM ^b^	*p*	SEM ^b^	*p*	SEM ^b^	*p*	SEM ^b^	*p*
*E. coli* O157 2017-5-590	0.0	0.0	0.0	0.0	0.0	-	-	-	-	-	-	-	-	-	-	-	-	-	-	-	-	-	-	-	-
*E. coli* O157 2017-5-493	0.0	0.0	0.0	0.0	0.0	-	-	-	-	-	-	-	-	-	-	-	-	-	-	-	-	-	-	-	-
*E.coli* O157 380-94	0.0	0.0	0.0	0.0	0.0	-	-	-	-	-	-	-	-	-	-	-	-	-	-	-	-	-	-	-	-
*C. jejuni* 2016-12-17A	0.0	0.0	4.1	5.8	11.1	0	1.00	−4.08	<0.0001	−5.75	<0.0001	−11.08	<0.0001	−4.08	<0.0001	−5.75	<0.0001	−11.08	<0.0001	−1.67	<0.0001	−7.00	<0.0001	−5.33	<0.0001
*C. coli* 2016-12-82A	1.4	3.2	3.8	7.0	12.7	−1.75	0.0001	−2.33	<0.0001	−5.58	<0.0001	−11.25	<0.0001	−0.58	0.1226	−3.83	<0.0001	−9.50	<0.0001	−3.25	<0.0001	−8.92	<0.0001	−5.67	<0.0001
*C. coli* 2016-12-80A	0.0	0.8	3.8	5.8	11.7	−0.75	0.0255	−3.75	<0.0001	−5.75	<0.0001	−11.67	<0.0001	−3.00	<0.0001	−5.00	<0.0001	−10.92	<0.0001	−2.00	<0.0001	−7.92	<0.0001	−5.92	<0.0001
*C. coli* 2016-12-181A	2.3	3.3	4.8	7.7	14.7	−1.083	0.1650	−2.50	0.0036	−5.42	<0.0001	−12.42	<0.0001	−1.42	0.0746	−4.33	<0.0001	−11.33	<0.0001	−2.92	0.0011	−9.92	<0.0001	−7.00	<0.0001
*S. Typhimurium* ATCC 14028	0.0	0.0	0.0	0.0	0.0	7.89 × 10^−31^	1.00	−7.89 × 10^−31^	1.00	0.00	1.00	2.31× 10 ^−15^	1.00	−1.58 × 10^−30^	1.00	−7.89 × 10^−31^	1.00	2.31 × 10^−15^	1.00	7.89 × 10^−31^	1.00	2.31 × 10^−15^	1.00	2.31 × 10^−15^	1.00
*S. aureus* VDL3-SA-2017	2.8	3.4	4.1	5.2	7.7	−0.63	0.0007	−1.29	<0.0001	−2.38	<0.0001	−4.88	<0.0001	−0.67	0.0004	−1.75	<0.0001	−4.25	<0.0001	−1.08	<0.0001	−3.58	<0.0001	−2.50	<0.0001
*P. aeruginosa* VDL4-PA-2017	0.0	0.0	0.0	0.0	0.3	1.11 × 10^−16^	1.00	2.22 × 10^−16^	1.00	1.67 × 10^−16^	1.00	−0.33	<0.0001	1.11 × 10^−16^	1.00	1.11 × 10^−16^	1.00	−0.33	<0.0001	5.55 × 10^−17^	1.00	−0.33	<0.0001	−0.33	<0.0001
*K. pneumoniae* VDL1-KP-2017	1.3	1.3	0.9	0.0	0.8	−0.083	0.6079	0.33	0.0512	1.25	<0.0001	0.42	0.0177	0.42	0.0177	1.33	<0.0001	0.50	0.0057	0.92	<0.0001	0.083	0.6079	−0.83	<0.0001
*K. oxytoca* VDL2-KO-2017	0.0	0.0	0.0	0.8	2.4	−4.44 × 10^−16^	1.00	4.44 × 10^−16^	1.00	−0.83	0.4575	−2.38	0.0441	8.88 × 10^−16^	1.00	−0.83	0.4575	−2.38	0.0441	−0.83	0.4575	−2.38	0.0441	−1.54	0.1771
*E. faecalis* ATCC 29212	1.7	2.3	3.0	3.4	6.5	−0.67	0.5496	−1.29	0.2527	−1.75	0.1268	−4.83	0.0003	−0.63	0.5746	−1.08	0.3348	−4.17	0.0013	−0.46	0.6800	−3.54	0.0045	−3.08	0.0113

^a^ ZOI: average zone of inhibition (diameter; mm). ^b^ SEM: standard error of the mean.

**Table 3:** Comparison of antimicrobial effects of different concentrations of sorghum phenolic extract vs. control.

	Sorghum Phenolic Extract Concentration (µg/mL)						Mean Differences with the *p-*Values									
	0	100	200	500	1000	4000	0.vs. 100		0.vs. 200		0.vs. 500		0.vs. 1000		0.vs. 4000	
Bacteria	ZOI ^a^	ZOI ^a^	ZOI ^a^	ZOI ^a^	ZOI ^a^	ZOI ^a^	SEM ^b^	*p*	SEM ^b^	*p*	SEM ^b^	*p*	SEM ^b^	*p*	SEM ^b^	*p*
*E. coli* O157 2017-5-590	0.0	0.0	0.0	0.0	0.0	0.0	-	-	-	-	-	-	-	-	-	-
*E. coli* O157 2017-5-493	0.0	0.0	0.0	0.0	0.0	0.0	-	-	-	-	-	-	-	-	-	-
*E.coli* O157 380-94	0.0	0.0	0.0	0.0	0.0	0.0	-	-	-	-	-	-	-	-	-	-
*C. jejuni* 2016-12-17A	0.0 ^e,f,g^	0.0	0.0	4.1 ^e^	5.8 ^f^	11.1 ^g^	0	1.0000	0	1.0000	−4.08	<0.0001	−5.75	<0.0001	−11.08	<0.0001
*C. coli* 2016-12-82A	0.0 ^h^	1.4 ^c^	3.2 ^d^	3.8 ^e^	7.0 ^f^	12.7 ^g^	−1.42	0.0010	−3.17	<0.0001	−3.75	<0.0001	−7.00	<0.0001	−12.67	<0.0001
*C. coli* 2016-12-80A	0.0 ^d,e,f,g^	0.0	0.8 ^d^	3.8 ^e^	5.8 ^f^	11.7 ^g^	−178 × 10^−^^17^	1.0000	−0.75	0.0255	−3.75	<0.0001	−5.75	<0.0001	−11.67	<0.0001
*C. coli* 2016-12-181A	0.0 ^h^	2.3 ^c^	3.3 ^d^	4.8 ^e^	7.7 ^f^	14.7 ^g^	−2.25	0.0076	−3.33	0.0003	−4.75	<0.0001	−7.67	<0.0001	−14.67	<0.0001
*S.* Typhimurium ATCC 14028	0.5	0.0	0.0	0.0	0.0	0.0	1.73	0.1004	1.73	0.1004	1.73	0.1004	1.73	0.1004	1.73	0.1004
*S. aureus* VDL3-SA-2017	0.0 ^h^	2.8 ^c^	3.4 ^d^	4.1 ^e^	5.2 ^f^	7.7 ^g^	−0.63	0.0007	−1.29	<0.0001	−2.38	<0.0001	−4.88	<0.0001	−0.67	0.0004
*P. aeruginosa* VDL4-PA-2017	0.0 ^h^	0.0 ^c^	0.0 ^d^	0.0 ^e^	0.0 ^f^	0.3 ^g^	1.083	<0.0001	1.083	<0.0001	1.083	<0.0001	1.083	<0.0001	0.75	<0.0001
*K. pneumoniae* VDL1-KP-2017	0.0 ^f^	1.3	1.3	0.9	0.0 ^f^	0.8	−0.1667	0.3101	−0.25	0.1346	0.17	0.3101	1.083	<0.0001	0.25	0.1346
*K. oxytoca* VDL2-KO-2017	0.0 ^g^	0.0	0.0	0.0	0.8	2.4 ^g^	1.78 × 10^−^^15^	1.0000	1.33 × 10^−^^15^	1.000	2.22 × 10^−^^15^	1.0000	−0.83	0.4575	−2.38	0.0441
*E. faecalis* ATCC 29212	0.0 ^d,e,f,g^	1.7	2.3 ^d^	3.0 ^e^	3.4 ^f^	6.5 ^g^	−1.52	0.1447	−2.13	0.0468	−2.71	0.0145	−3.13	0.0058	−5.95	<0.0001

^a^ ZOI: average zone of inhibition (diameter; mm). ^b^ SEM: standard error of the mean. ^c^ evidence of statistical difference between 0.vs. 100 µg/mL at α = 0.05. ^d^ evidence of statistical difference between 0.vs. 200 µg/mL at α = 0.05. ^e^ evidence of statistical difference between 0.vs. 500 µg/mL at α = 0.05. ^f^ evidence of statistical difference between 0.vs. 1000 µg/mL at α = 0.05. ^g^ evidence of statistical difference between 0.vs. 4000 µg/mL at α = 0.05. ^h^ evidence of statistical difference between all sorghum phenolic extract concentrations at α = 0.05.

## Data Availability

Data is contained within the article.
